# The Role of Multimodality Imaging in the Diagnosis and Follow-Up of Malignant Primary Cardiac Tumors: Myxofibrosarcoma—A Case Report and Literature Review

**DOI:** 10.3390/diagnostics13101811

**Published:** 2023-05-20

**Authors:** Adela Șerban, Alexandra Dădârlat-Pop, Raluca Tomoaia, Cătălin Trifan, Adrian Molnar, Simona Manole, Alexandru Achim, Mihai Suceveanu

**Affiliations:** 1 Cardiology Department, Heart Institute Niculae Stăncioiu, 19-21 Motilor Street, 400001 Cluj-Napoca, Romania; 2 5th Department of Internal Medicine, Iuliu Haţieganu University of Medicine and Pharmacy, 8 Victor Babes Street, 400012 Cluj-Napoca, Romania; 3 Clinical Rehabilitation Hospital, 46-50 Viilor Street, 400347 Cluj-Napoca, Romania; 4 Cardiovascular Surgery Department, Heart Institute Niculae Stăncioiu, 19-21 Motilor Street, 400001 Cluj-Napoca, Romania; 5 Radiology Department, Heart Institute Niculae Stăncioiu, 19-21 Motilor Street, 400001 Cluj-Napoca, Romania

**Keywords:** cardiac tumors, myxofibrosarcoma, multimodality imaging

## Abstract

Cardiac tumors are a very rare but heterogenous group of diseases that may reveal themselves through a variety of nonspecific cardiac symptoms that may pose a challenge to the diagnostic process. Myxofibrosarcoma is a particularly rare type of cardiac tumor that carries a poor prognosis, thus making accurate and timely diagnosis essential. A 61-year-old woman presented with fatigue and shortness of breath during mild exercise, symptoms that have progressively worsened during the previous year. Multimodality imaging consisting of transthoracic and transesophageal echocardiography (TTE and TEE), cardiac magnetic resonance (CMR), cardiac computer tomography (CCT), and fluorodeoxyglucose positron emission computer tomography (18F-FDG PET-CT) was used for the diagnosis and postoperative follow-up of a myxofibrosarcoma.

## 1. Introduction

Cardiac tumors are extremely rare findings, which can involve any of the heart structures. They are classified as primary and secondary (metastatic heart tumors). Primary cardiac tumors’ (PCTs) prevalence is only 0.001–0.03%, according to most autopsy series, and, even if secondary cardiac tumors may be up to 40 times more frequent, they still represent an uncommon diagnosis [1]. The vast majority of the PCTs are benign, 75–90%, 50% of which being represented by myxomas. [2,3] Malignant PCTs account for around 5–25% of the PCTs and are represented by sarcomas, lymphomas, and mesotheliomas [4,5]. The World Health Organization’s 2015 classification of tumors of the heart and pericardium included, in addition to benign and malignant PCTs, tumors of uncertain biological behavior (inflammatory myofibroblastic tumor and paraganglioma) and germ cell tumors (teratoma, yolk sac tumor). Secondary cardiac tumors (SCTs) usually originate from melanomas; lung, breast, and renal cancers; or lymphomas and may occur in up to 9–18% of oncologic patients [1,6,7].

Myxofibrosarcoma (MFS) represents one of the least frequent cardiac sarcomas. Histologically, it is defined as being composed of spindle cells and polygonal cells with atypic nuclei with a myxoid or fibrous background [6,8]. The most frequent site of this type of tumor is represented by the left atrium, but it was also described in the other heart chambers [3]. Usually, the patients are asymptomatic until the tumor reaches significant dimensions or diffuses either locally or by metastases. The patients may develop congestive heart failure symptoms, syncope, thromboembolism, or arrhythmias, depending on the location and size of the tumor as well as the infiltration of the adjacent tissues that may cause obstruction of the right or left outflow tracts, disturbances of the conduction system, or pericardial effusion [1,9,10].

MFS is a particularly aggressive type of cancer, in which timely surgical resection and chemotherapy are essential. The importance of complete tumor resection cannot be overstated, as the available data show significant differences in the median survival time of patients with complete resection (53.5 months) as opposed to those with incomplete resection (9.5 months) [5]. These aspects underscore the need for a swift and accurate diagnosis using proper diagnostic algorithms, as well as the distinctive value of an early timed surgical intervention. By presenting this case report, we aim at raising awareness regarding the crucial steps of performing a quick and accurate differential diagnosis of a cardiac tumor that may improve survival in patients with malignant primary cardiac tumors.

## 2. Case Presentation

A 61-year-old woman, hypertensive, with a sole episode of atrial flutter spontaneously converted to sinus rhythm 2 months prior to the admission, who underwent an excisional right breast surgery of a benign tumor 19 years ago, presented with fatigue and shortness of breath during mild exercise, symptoms that have progressively worsened over the previous 12 months. In addition, the patient described recurring episodes of rapid, irregular palpitations with a duration of less than 10 s. The physical exam showed normal blood pressure (110/70 mmHg), heart rate (65/min), and blood oxygen saturation (97% in room air); rhythmic heartbeats, with a harsh crescendo-decrescendo pulmonic stenosis murmur radiating to the entire heart auscultation area; and slight lower limb edema, with no other pathological findings. An ECG observed sinus rhythm, low cardiac voltage in the limb leads, and negative T waves in the V1-V4 leads. NT-pro-BNP levels were moderately elevated (549.7 pg/mL), but D-Dimer was normal (196 ng/mL), and no inflammatory syndrome, anemia, renal or hepatic abnormalities were found. A chest x-ray was within limits. Given the high thrombo-embolic stroke risk (CHA_2_DS_2_-VASc score = 3-female hypertensive with preserved ejection fraction heart failure) and low bleeding risk (HAS-BLED score = 0), the patient was under anticoagulant therapy with Apixaban 5 mg, twice daily.

**Figure 1:**
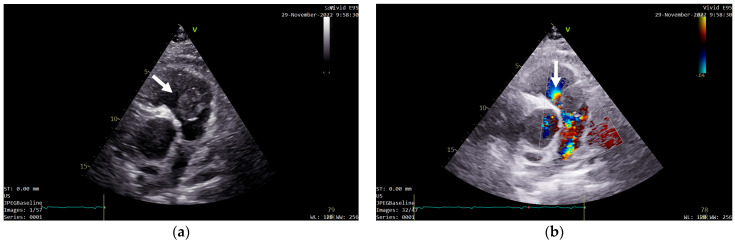
Transthoracic echocardiography (TTE) showing: (**a**) a round intracardiac mass attached to the right ventricle outflow tract (RVOT) wall by a pedunculus (white arrow); (**b**) turbulent flow on Color Doppler caused by the tumor’s severe obstruction of the RVOT (white arrow).

**Figure 2:**
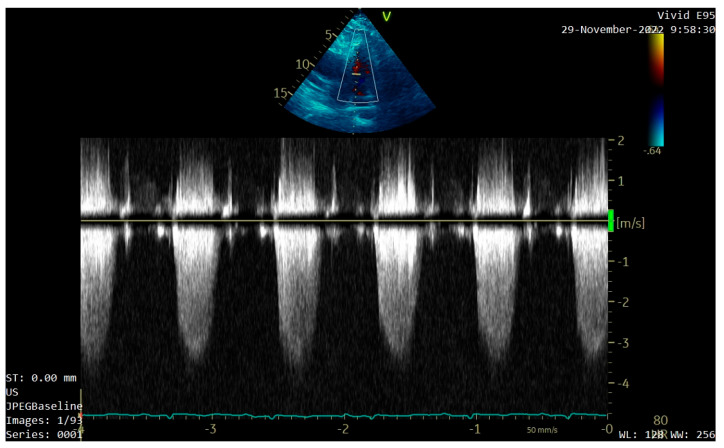
TTE Continuous Wave Doppler showing severe RVOT obstruction.

A transthoracic echocardiography (TTE) was performed. It showed a round 25/30 mm, 6.5 cm^2^ intracardiac mass attached to the right ventricle outflow tract (RVOT) wall by a 5 mm thick pedunculus (Figure 1a), which prolabated through the pulmonary valve in systole, thus causing severe obstruction at this level with a maximum gradient of 70 mmHg (Figure 1b and Figure 2). The pulmonary valve itself presented no structural abnormalities, but the right ventricle was dilated (50 mm at the base) with a slightly decreased systolic function (TAPSE = 15 mm), a moderate tricuspid regurgitation and paradoxical interventricular septum motion being also observed due to right ventricular pressure overload. The left ventricle was not dilated and had a preserved systolic function, and only a mild mitral regurgitation was noted.

**Figure 3:**
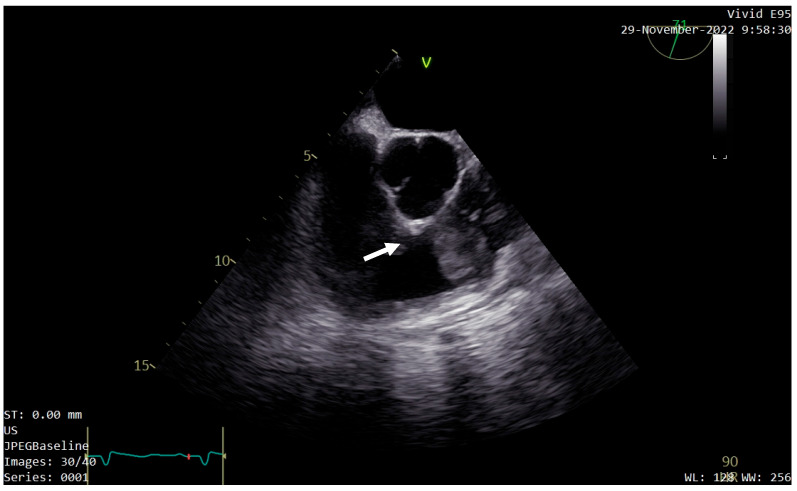
Transesophageal echocardiography (TEE) confirming the location, size, and shape of the tumor (white arrow).

For a better evaluation of the tumor, transesophageal echocardiography (TEE) was performed. It confirmed the location, size, and shape of the tumor, describing it as having low echogenicity, with a similar texture to the myocardium (Figure 3). Comparable pressure gradients at the pulmonary valve were recorded, and patent foramen ovale was identified.

**Figure 4:**
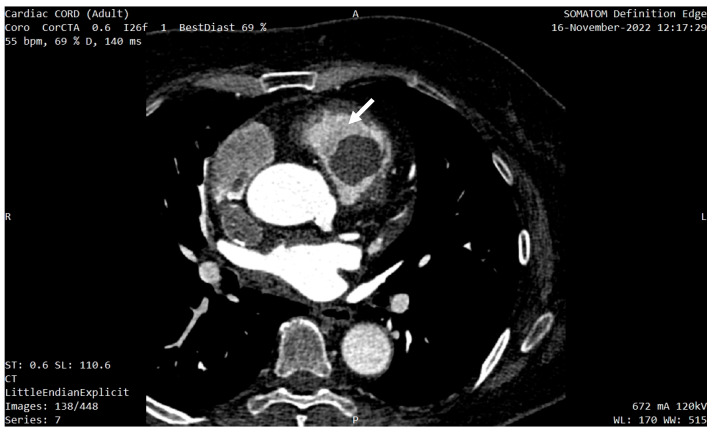
Cardiac CT showing the mass localization 3 mm anterior of the pulmonary valve (white arrow), occupying 80% of the RVOT.

**Figure 5:**
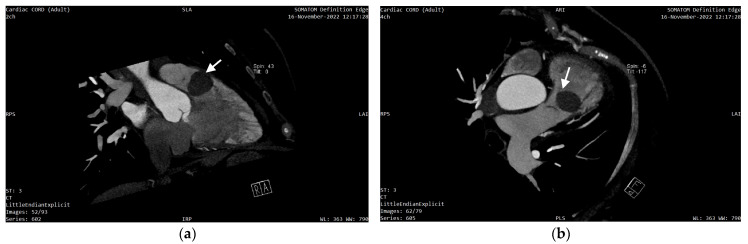
Cardiac CT reconstruction showing tumor (white arrow) correspondence to the neighboring anatomical structures: (**a**) two chambers reconstruction; (**b**) four chambers reconstruction.

In order to better characterize the tumor, the investigations were completed with a cardiac CT and a cardiac MRI (CMR). The CT described a 33/26/23 mm hypodense, oval-shaped, slightly lobulated, well-circumscribed mass located 3 mm anterior of the pulmonary valve, which occupied 80% of the RVOT and was in contact with the medial and anterior wall of the RV (Figure 4 and Figure 5a,b). No coronary artery lesions were found.

**Figure 6:**
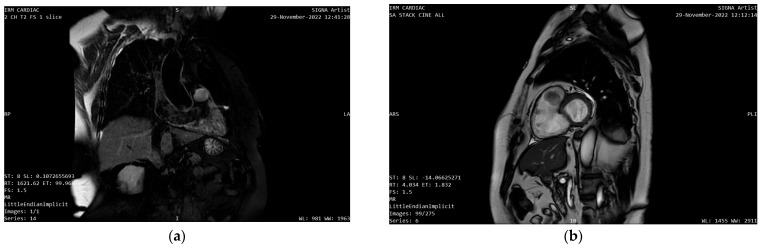
Cardiac MRI (CMR) showing the mass’ (white arrow): (**a**) high signal intensity on the fat suppressed T2 weighted sequence; (**b**) late contrast enhancement.

The CMR showed a 17/23/21 mm round-oval shaped mass situated in the RVOT infundibulum, just below the pulmonary valve, which was causing RVOT obstruction. The mass had low T2 signal intensity, and high signal intensity on the fat suppressed T2 weighted sequence (Figure 6a), with late contrast enhancement (Figure 6b). An 8 mm mass pedunculus allowing ample tumor motion was observed. It appeared to be attached to the anterolateral infundibulum wall with a slight thickening and contrast enhancement on a 10 mm portion of the wall, suggesting a diagnosis of myxoma with adherent thrombi.

Considering the multimodal imaging results, a first probable diagnosis of myxoma was established.

Given the significant obstruction in the RVOT caused by the tumor, the high embolic risk, and in order to decide upon the best therapeutic course of action, the case was discussed in the Heart-Team, which recommended the surgical excision of the tumor.

After mid-line sternotomy and establishment of routine cardiopulmonary bypass, both vena cavae were snared, and, on a beating heart, the right atrium was opened with a vertical incision. On inspection, there was no involvement of the right atrium, and the tricuspid valve appeared normal, with good coaptation. Two retractors were used to expose the right ventricular chamber through the tricuspid valve. A 3/3 cm mass with high mobility, attached through a small pedunculus to the RVOT anterior wall and causing complete occlusion of the RVOT, was found. Using scissors and diathermy, the tumor was excised along with a small portion of cardiac muscle, which resulted in a small opening in the RV free wall that was, subsequently, repaired using a 3–0 polypropylene suture. The pulmonary valve was also inspected and appeared to be normal and having good coaptation. The right atrium was closed, and the patient was easily weaned from cardiopulmonary bypass with no inotropic of vasopressor support being required. Hemostasis was achieved, and the sternotomy wound was closed. The postoperative period was uneventful, and the immediate echocardiographic control observed slightly dilated RV (45 mm at the base), with preserved systolic function, moderate tricuspid regurgitation, and no obstruction in the RVOT. During the hospitalization, the patient did not continue to present palpitations, and no atrial flutter or other rhythm disturbances were observed. However, considering the high stroke risk as well as the low bleeding risk, the anticoagulant therapy with Apixaban 5 mg twice daily was continued, and the patient was recommended periodical Holter-ECG monitoring.

**Figure 7:**
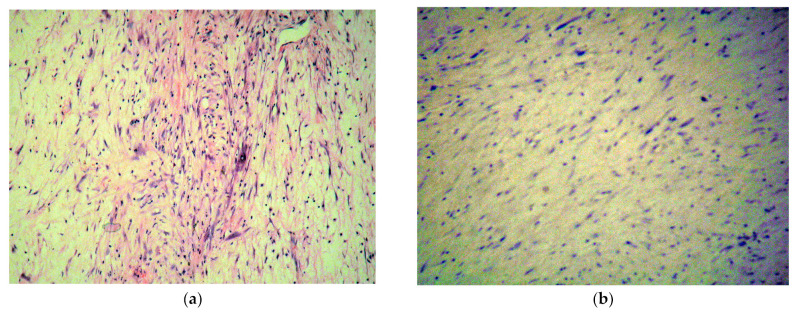
(**a**) Hematoxylin-Eosin staining showing mesenchymal proliferation composed of fasciculate and myxoid areas containing cells with elongated and stellate nuclei. (**b**) Immunohistochemistry staining with negative result for calretinin, thus infirming the initial myxoma diagnosis/lack of typical brown colored areas.

The pathology report described a mesenchymal proliferation composed of fasciculate and myxoid areas containing cells with elongated and stellate nuclei (Figure 7a), with extremely rare mitoses and a variable proliferation index of 10–20%, with positive resection margins. Focal necrosis was found in 10–15% of the tumor. Surprisingly, the immunochemistry showed positive actin, rarely positive desmin, inconclusive neuron specific enolase, negative CD31 and CD34, negative cytokeratin, negative calretinin (Figure 7b), and S100. The above-mentioned findings did not support the cardiac myxoma diagnosis but suggested a G1 low grade myxofibrosarcoma.

The patient was referred to an oncology specialist, who recommended chemotherapy initiation. Chemotherapy with Gemcitabine and Docetaxel was started.

**Figure 8:**
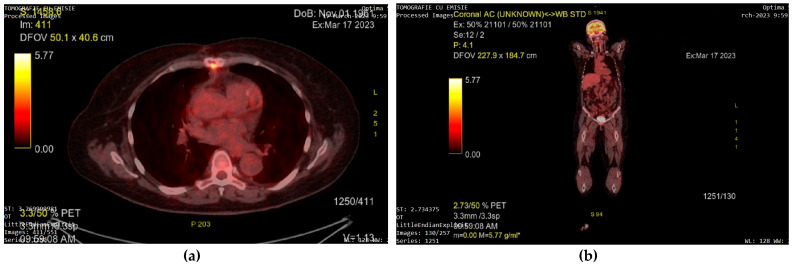
18F-FDG fluorodeoxyglucose (18F-FDG) PET-CT showing: (**a**) an encapsulated effusion adjacent to the right ventricle infundibulum (white circle) with minimum calcification, thin minimally active margins, and no other 18F-FDG fixation zones at this level; slight 18F-FDG fixation at the sternum level due to recent sternoraphy; (**b**) no distant metastases on the whole-body examination.

Follow-up whole-body PET-CT was performed 3 months after the surgical intervention. Adjacent to the right ventricle infundibulum, a 30/19 mm area corresponding to the pericardium was observed. It presented minimum calcification, thin minimally 18F-FDG fluorodeoxyglucose (18F-FDG) active margins, and no other fixation zones (Figure 8a). Considering the modest 18F-FDG fixation, the area was interpreted as an encapsulated effusion, and CMR reevaluation along with periodical PET-CT monitoring of the lesion was recommended. Slight 18F-FDG fluorodeoxyglucose (18F-FDG) fixation was also observed at the sternum level, given the recent sternoraphy, but no distant metastases were found (Figure 8b).

## 3. Discussion

Timely diagnosis and swift therapeutical actions are essential for improving patient survival in MFS and in malignant PCTs in general, as they carry a poor prognosis, in contrast to the lack of symptoms defining the early stages of the disease. Given our patient’s breast tumor history, it is worth mentioning that secondary cardiac tumors (SCTs) are 20–40-fold more frequent than PCTs, and about 10–12% of them are breast cancer metastases [1,10,11]. Second only to lung cancer and tied to hematologic malignancies, breast cancer represents the 2nd most common SCT origin [10]. This information may prove valuable during differential diagnosis, as it shows greater than expected SCT incidence in selected groups of patients. The data from literature show SCTs occurring even long after the initial cancer diagnosis and treatment (more than 20 years), either after confirmed cancer relapse or as a first sign of cancer recurrence [7,12,13]. Confirmed metastases in other territories raises the likelihood of SCTs from 9% to 18%, according to available data [7]. However, this is not the case for our patient, as the pathology report concluded that the breast tumor diagnosed 19 years ago was benign.

In addition to systemic manifestations (fever, weight loss, arthralgias, fatigue, paraneoplastic syndromes), pulmonary embolism, or systemic embolism, cardiac tumors may cause cardiac manifestations, including interference with myocardial function and blood flow, pericardial effusion, or cardiac arrhythmias [1,9,10]. Cardiac arrhythmias caused by cardiac tumors are relatively diverse and may be particular to the site of involvement and tumor type. There are no specific data on myxofibrosarcoma, but, in general, most frequent arrhythmias attributed to cardiac tumors include atrial premature contractions, atrial tachycardia, atrial flutter, atrial fibrillation, Wolff–Parkinson–White syndrome, ventricular premature contractions, ventricular tachycardia, torsades de pointes, and AV blocks [14]. Our patient’s history included one episode of atrial flutter. However, the tumor localization in the RVOT madeit unlikely to be responsible for this arrhythmia. Nevertheless, the patient was recommended to continue the anticoagulant therapy following the surgery, as it had a high stroke risk and a low bleeding risk, in addition to the recently discovered malignancy. This indication may be reviewed according to periodical Holter-ECG monitoring results and patient symptoms, as the atrial flutter documented before the surgery was highly symptomatic. It should be noted that the patient may develop atypical flutter secondary to the surgical atrial scar.

Echocardiography has a high sensitivity (90%) and specificity (95%) for detecting cardiac tumors [1]. Transthoracic (TTE) and transesophageal (TEE) techniques can supply valuable information regarding the shape, size, extent, mobility, and location of the tumor and evaluate its hemodynamic impact. Echocardiography is optimal for evaluating small mobile masses due to its high temporal and spatial resolution. TEE may be more precise than TTE in detecting very small tumors (<5 mm), or tumors found in the posterior cardiac segments, as well as evaluating patients with chronic lung disease or obesity [3,9,15,16]. Use of echocardiographic contrast agents may be beneficial in confirming an intracardiac mass in patients with poor acoustic window, as well as in the differential diagnosis with thrombi or between benign and malignant tumors, the latter having better vascularization, even when compared to the myocardium. Three-dimensional echo may offer additional information on the shape, size, volume, and mobility of a tumor, offering a more accurate evaluation through 3D reconstruction [9,15,16,17]. In patients who are Cardiac Magnetic Resonance (CMR) eligible, the CMR can provide useful data regarding tumor histopathology (calcification, fat infiltration, necrosis, fibrosis, fluid, hemorrhage within the tumor) and may more accurately describe the tumor location, extension, mobility, and valvular implication [2,3,9,18]. Given its outstanding spatial resolution, Cardiac CT (CCT) can accurately evaluate the tumor’s relationship with the cardiac layers and structures (myocardium, pericardium, valves) and other non-cardiac structures located in the chest (lungs, vascular structures) [2,3,9]. The MFS Cardiac Magnetic Resonance (CMR) and Cardiac CT (CCT) evaluation is characterized by non-specific features, usually describing a heterogenous, infiltrative mass. Differential diagnosis needs to exclude thrombi and broad-based myxoma, which are more common. Invasive behavior such as pulmonary vein extensions or pericardium infiltrations support the diagnosis of myxofibrosarcoma [2,9]. Complete evaluation of a cardiac tumor can be obtained by performing positron emissions tomography (PET), which offers information regarding the metabolic activity of the tumor using fluorodeoxyglucose (18F-FDG). 18F-FDG PET scanning has been shown to have 100% sensitivity and 92% specificity in differentiating benign and malignant cardiac masses (with malignant tumors having a relatively high 18F-FDG uptake compared to none or just slight uptake in benign tumors in general). PET can also be a useful tool in the staging, treatment, and prognosis of cardiac tumors, as well as in assessing early cancer therapy responses [9]. Consistent with the case of Reddy et al. [4], we used 18F-FDG PET as a prognostic tool after surgical resection to search for any metastatic foci during short- and long-term follow-up.

There are just a few cases in the literature using multimodality imaging, but, when available, we consider that all imaging methods should be used. As in the case of our patient, not only for the correct diagnosis but also for selecting the optimal surgical approach, each imaging technique contributes to a better characterization of a cardiac tumor and its relationship with the neighboring structures. This statement is supported by the work of Tyebally et al. [3] and Paraskevaidis et al. [1], which have included all the above-mentioned imaging modalities in their diagnostic approach of a cardiac mass. As our patient has also benefited from this algorithm, proving its validity once again, we encourage healthcare stakeholders to work towards increasing the availability of multimodality imaging.

The cornerstone treatment of myxofibrosarcoma is represented by excisional surgery. Successful complete resection of the tumor greatly improves the patient survival rate, with patients having a five-fold longer survival rate than in the case of incomplete resection [5,19]. Unfortunately, complete resection is difficult to attain due to tumor extent and localization and is reportedly achieved in only around 30% of the cases [20]. Sadly, this was the case of our patient, whose tumor resection margins were positive. However, the 3-month postoperative follow-up PET-CT of our patient showed no local or distant tumor recurrence, and this aspect may weigh in on reconsidering an improved prognosis. Chemotherapy and radiotherapy may contribute to improving the survival rate, though literature data are sparse given the rarity of this type of malignant tumor [1,3]. We acknowledge that a multimodality imaging approach may not be readily available in every center, and, considering the importance of a timely surgical resection, this should not be a cause of delay. A list of reviewed MFS articles including a brief comparison of relevant diagnostic and therapeutic characteristics is depicted in Table 1.

## 4. Conclusions

In conclusion, considering that myxofibrosarcoma is a very rare malignant cardiac tumor that can present itself in various forms and carries a poor prognosis, multimodality imaging is essential for correct diagnosis and optimal surgical planning. In addition, multimodality imaging can be a useful tool in assessing the response to oncologic treatment during follow-up.

## Figures and Tables

**Table 1:** List of reviewed myxofibrosarcoma (MFS) articles and comparison of relevant diagnostic and therapeutic characteristics.

Title	Author	Patient Age	MFS Localization	Imaging Technique Used	Complete Surgical Resection	Oncologic Treatment	Survival Time
Primary cardiac myxofibrosarcoma: case report, literature reviewand pooled analysis [5]	Sun D et al.	41 (mean)	Left atrium (predominant)	Chest X-Ray, TTE, TEE	83%	Chemotherapy (various regimens)Radiotherapy	32 months (mean)
A case report of primary cardiac myxofibrosarcoma presenting with severecongestive heart failure [21]	Uhijira K et al.	29	Left atrium	TTE, CCT	No	Radiotherapy	>12 months
Low grade myxofibrosarcoma in the right ventricle presenting as pulmonary thromboembolism [20]	Suh JH et al.	78	Right ventricle	TTE, CCT	No	NA	NA
Primary cardiac myxofibrosarcoma with osteoid differentiation mimicking a left atrial myxoma: A rare entity [4]	Reddy KVC et al.	62	Left atrium	Chest X-Ray, TTE, TEE, CMR, 18-FDG PET-CT	Yes	Chemotherapy (doxorubicin, ifosfamide) Radiotherapy	>12 months
Multimodality imaging in cardiac Myxofibrosarcoma [18]	Li X et al.	55	Pericardium	TTE, CCT, CMR	Yes	NA	NA

MFS, Myxofibrosarcoma; TTE, Transthoracic echocardiography; TEE, Transesophageal echocardiography; CCT, Cardiac Computer Tomography; CMR, Cardiac Magnetic Resonance; 18-FDG PET-CT–18 fluorodeoxyglucose positron emission tomography-computed tomography; NA, not applicable.

## Data Availability

Not applicable.

## References

[1] Paraskevaidis I.A., Michalakeas C.A., Papadopoulos C.H., Anastasiou-Nana M. (2011). Cardiac tumors. ISRN Oncol..

[2] Hoey E., Mankad K., Puppala S., Gopalan D., Sivananthan M. (2009). MRI and CT appearances of cardiac tumours in adults. Clin. Radiol..

[3] Tyebally S., Chen D., Bhattacharyya S., Mughrabi A., Hussain Z., Manisty C., Westwood M., Ghosh A.K., Guha A. (2020). Cardiac Tumors: JACC Cardio Oncology State-of-the-Art Review. JACC Cardio Oncol..

[4] Reddy K.C., Kumar P., Sanzgiri P., George A. (2020). Primary cardiac myxofibrosarcoma with osteoid differentiation mimicking a left atrial myxoma: A rare entity. J. Cardiol. Cases.

[5] Sun D., Wu Y., Liu Y., Yang J. (2018). Primary cardiac myxofibrosarcoma: Case report, literature review and pooled analysis. BMC Cancer.

[6] Burke A., Tavora F. (2016). The 2015 WHO Classification of Tumors of the Heart and Pericardium. J. Thorac. Oncol..

[7] Lichtenberger J.P., Reynolds D.A., Keung J., Keung E., Carter B.W. (2016). Metastasis to the Heart: A Radiologic Approach to Diagnosis with Pathologic Correlation. AJR Am. J. Roentgenol..

[8] Liu H., Zhang X., Zhang S., Yu S. (2021). Analysis of prognostic factors in 171 patients with myxofibrosarcoma of the trunk and ex-tremities: A cohort study. Ann. Transl. Med..

[9] Lin Y., Wu W., Gao L., Ji M., Xie M., Li Y. (2022). Multimodality Imaging of Benign Primary Cardiac Tumor. Diagnostics.

[10] Goldberg A., Blankstein R., Padera R.F. (2013). Tumors Metastatic to the Heart. Circulation.

[11] Osmani A.H., Suleman K. (2021). Breast Cancer with Metastases to Coronary Arteries. J. Coll. Physicians Surg. Pak..

[12] Chou W.-H., Chi N.-H., Wang Y.-C., Huang C.-H. (2016). Metastatic breast cancer with right ventricular erosion. Eur. J. Cardio-Thoracic Surg..

[13] Xu F., Wang X., Jiang Z. (2019). A right atrium metastasis of breast cancer after long-term endocrine therapy. Ann. Transl. Med..

[14] Jayaprakash S. (2018). Clinical presentations, diagnosis, and management of arrhythmias associated with cardiac tumors. J. Arrhythmia.

[15] Mankad R., Herrmann J. (2016). Cardiac tumors: Echo assessment. Echo Res. Pract..

[16] L’Angiocola P.D., Donati R. (2020). Cardiac Masses in Echocardiography: A Pragmatic Review. J. Cardiovasc. Echogr..

[17] Lanzoni L., Bonapace S., Dugo C., Chiampan A., Anselmi A., Ghiselli L., Molon G. (2022). Cardiac Masses and Contrast Echocardiography. Eur. Heart J. Cardiovasc. Imaging.

[18] Li X., Jiang Y., Zhao R., Yu Y. (2022). Multimodality imaging in cardiac myxofibrosarcoma. Eur. Hear. J. Case Rep..

[19] Castronovo C., Arrese J.E., Quatresooz P., Nikkels A. (2013). Myxofibrosarcoma: A Diagnostic Pitfall. Rare Tumors.

[20] Suh J.H., Kim D.Y., Yoon J.S., Park E.S., Park C.B. (2017). Low grade myxofibrosarcoma in the right ventricle presenting as pulmonary thromboembolism. J. Thorac. Dis..

[21] Ujihira K., Yamada A., Nishioka N., Iba Y., Maruyama R., Nakanishi K., Shimizu A., Hatanaka K.C., Mitsuhashi T., Shinohara T. (2016). A case report of primary cardiac myxofibrosarcoma presenting with severe congestive heart failure. J. Cardiothorac. Surg..

